# Load-bearing capacity and Weibull characteristics of inlay-retained fixed dental prosthesis: Effect of zirconia type, bonded substrate and aging

**DOI:** 10.1007/s00784-026-07147-5

**Published:** 2026-09-24

**Authors:** Meret Helbling, Luiza Freitas Brum Souza, Abdülhakim Alpaslan Sener, Enkelejd Angelo Cankja, Tan Fırat Eyüboğlu, Mutlu Özcan

**Affiliations:** 1 Center for Dental Medicine, Clinic of Masticatory Disorders and Dental Biomaterials, University of Zurich, Plattenstrasse 11, Zurich, CH-8032 Switzerland; 2 Faculty of Dentistry, Department of Endodontics, Istanbul Medipol University, Istanbul, Türkiye Turkey

**Keywords:** Adhesion, Dental materials, Prosthetic dentistry, Thermomechanical aging, Weibull analysis, Zirconia

## Abstract

**Objectives:**

This study evaluated the load-bearing capacity and reliability of different zirconia materials used for *inlay-retained fixed dental prostheses (IRFDPs)*, considering the influence of substrate type (tooth substrate or resin composite) and thermo-mechanical aging.

**Materials and methods:**

IRFDPs (*N* = 160) were fabricated using four zirconia materials: IPS e.max ZirCAD LT (3Y-TZP), Katana YML (3Y-5Y graded zirconia), Katana HTML Plus, and Katana STML (*n* = 40 each). Restorations were adhesively cemented onto tooth or resin composite substrates and tested either under dry conditions or after thermo-mechanical aging (1,200,000 cycles, 50 N, 5–55 °C). Fracture load was determined using monotonic load-to-fracture testing. Data were analyzed using two-way ANOVA and Tukey’s test (α = 0.05). Reliability of durability was assessed using Weibull analysis.

**Results:**

Under dry conditions, material (*p* = 0.0047), substrate (*p* = 0.0237), and their interaction (*p* = 0.0004) significantly influenced fracture load. YML-composite (1406 ± 386 N) and HTML-composite (1142 ± 249 N) showed higher values than STML-composite (553 ± 246 N). No significant differences were observed on tooth. substrate After aging, material and interaction effects remained significant (*p* < 0.05). HTML-tooth showed the highest fracture load (1727 ± 351 N), while STML-composite showed the lowest (718 ± 292 N). Weibull analysis indicated comparable reliability after aging.

**Conclusions:**

Zirconia type and substrate significantly influenced mechanical performance of IRFDPs. High-translucent and graded zirconia showed substrate-dependent behavior, whereas 3Y zirconia was more stable.

**Clinical relevance:**

Zirconia selection and bonding substrate influence fracture resistance of IRFDPs. High-translucent zirconia requires careful substrate selection, while 3Y zirconia provides more consistent performance in posterior applications.

## Introduction

 Replacing missing posterior teeth is challenging in restorative dentistry. Traditional treatments include single crowns on implants or fixed dental prostheses (FDPs) with full-coverage crowns, though these involve removing significant dental tissue and risk pulp damage [1]. When enough healthy tooth structure remains and implant is not indicated, inlay-retained fixed dental prostheses (IRFDPs) offer a less invasive alternative, preserving more natural tissue and reducing biological risks [1–3]. IRFDPs are suitable when abutment teeth have sufficient residual structure, lowering pulp damage risk [1, 3]. Their success depends on material choice, preparation, load distribution, and bond quality [2, 3].

Fractures and debonding issues with lithium disilicate IRFDPs led to the use of veneered zirconia, which often chipped, affecting durability [2, 4]. Monolithic zirconia, which lacks veneering, reduces chipping risk and supports minimally invasive procedures [5]. High strength, toughness, and biocompatibility of zirconia, made this material a common choice in restoring missing teeth during the last two decades. Early zirconia type was opaque, requiring veneering for aesthetics [6], but increased yttria enhanced translucency at the cost of strength and toughness [7]. About 4 mol% yttria zirconia offers better aesthetics but less fatigue resistance, exemplifying the trade-off between translucency and mechanical performance.

Multilayer zirconia materials improve aesthetics by incorporating polychromatic and translucent layers within a single disc, mimicking natural dentin-enamel transitions [8–10]. These materials can show optical or compositional gradients with varied amounts of yttria. These materials can exhibit either optical gradients or compositional gradients with varying yttria concentrations across layers [6]. Pöppel et al. found that yttria content influences fracture resistance in that higher yttria reduces strength, and layer thickness and connector size are critical for overall strength [7]. Flexural strength, assessed with Weibull statistics, reflects material reliability, with higher Weibull modulus indicating more homogeneous and reliable material [10, 11]. Adhesive bonding sensitivity affects IRFDP durability, with debonding a common failure mode [2, 4]. Surface pretreatment, including airborne-particle abrasion and MDP primers, enhances zirconia bonding [6]. Bonding to enamel is predictable, but hydrophilic nature of dentin and dentinal tubules challenge adhesive durability, especially with aging. Bond strength studies show higher strength on composite than dentin, underscoring substrate effects on adhesion [12].

Although multilayer zirconia materials are increasingly used for their esthetic appeal, evidence on their mechanical behavior in IRFDPs is limited. The combined effects of zirconia type, bonded substrate, and aging on IRFDP load capacity and reliability remain unclear. There is no clear consensus on how different zirconia types perform when bonded to tooth or resin composite, or how aging affects their load capacity and reliability. Understanding the interaction among zirconia, substrate, and aging is crucial for optimizing material choice and the long-term success IRFDPs. Therefore, this study compared three multilayer zirconia materials (Katana HTML, Katana YML, Katana STML; Kuraray Noritake) with traditional monolithic zirconia (IPS e.max ZirCAD LT; Ivoclar) used as IRFDPs. The objective was to assess how material type, bonded substrate (tooth substrate versus resin composite), and artificial aging affect load capacity and Weibull characteristics. The null hypotheses tested were: (1) load capacity and Weibull characteristics among zirconia types would not show significant difference; (2) bonded substrate type would not influence the load-bearing capacity and Weibull characteristics of IRFDPs; and (3) aging would not have significant effect.

## Materials and methods

### Experimental model preparation

Caries-free human third molars (*N* = 320) were collected following extraction at the Center for Dental Medicine (ZZM) of the University of Zurich for research and other purposes. Third molars were selected to provide a sufficient number of caries-free teeth for standardized preparation. Each tooth had preauthorization from the respective donors in the form of written informed consent for secondary use, in accordance with directives set by the National Federal Council [13]. Ethical guidelines [14] were strictly followed, and irreversible anonymization was performed in accordance with State and Federal Law [15, 16]. After extraction, the teeth were stored in 0.5% chloramine T solution at 5 °C until use.

**Fig. 1:**
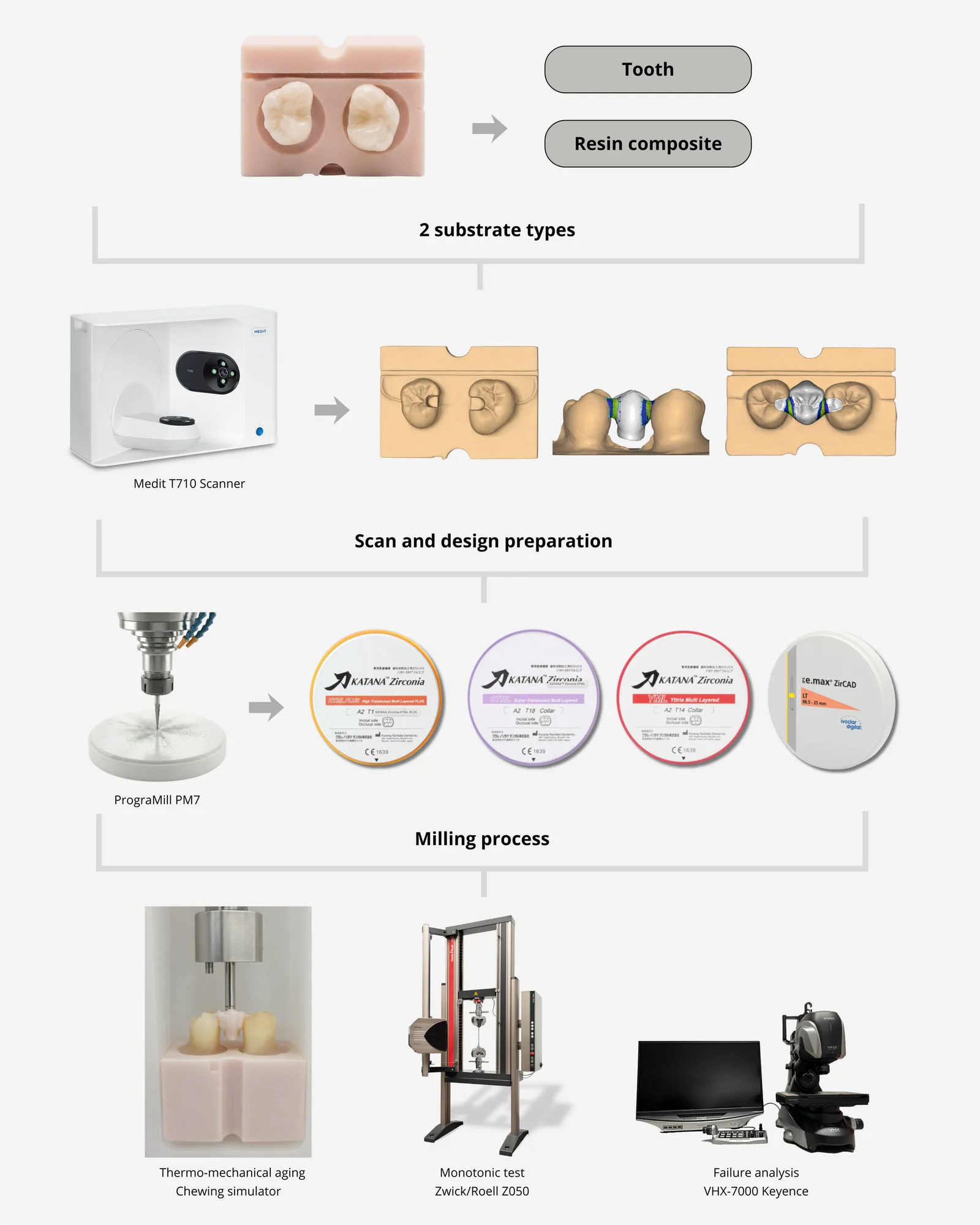
Experimental procedures

Experimental models (*N* = 160) were fabricated using a 3D-printing system. Each model contained two cylindrical cavities to accommodate two extracted molars. The teeth were embedded in the cavities with an auto-polymerized polymethyl methacrylate resin (Technovit 4071, Heraeus Kulzer, Germany) in accordance with the manufacturer’s instructions. Each model simulated a premolar-molar configuration **(**Fig. 1**)**.

A standardized inter-abutment distance of 7 mm was set to represent a missing premolar site. A custom-designed 3D-printed positioning template ensured precise alignment and spacing of the embedded teeth. The inter-abutment distance was verified with a digital caliper (± 0.01 mm accuracy). During specimen preparation, the models were stored in 0.5% chloramine T solution to prevent dehydration.

### Substrate preparation

Standardized Class II cavity preparations were defined before specimen preparation. For premolars (*n* = 160), cavity dimensions were standardized to 2.7 mm bucco-lingual width and 2.7 mm mesio-distal width, with the cervical margin positioned 1 mm above the cement-enamel junction. For molars (*n* = 160), cavity dimensions were standardized to 3.4 mm bucco-lingual width and 3.8 mm mesio-distal width, with the cervical margin likewise positioned 1 mm above the cemento-enamel junction.

A single operator performed all cavity preparations using a high-speed handpiece with continuous water-spray cooling. Initial cavity outlines were prepared with Intensiv Cerinlay burs (#011, Intensiv SA, Montagnola, Switzerland). Final cavity refinement was performed with diamond finishing burs (Komet SFD7; sizes 1 and 2, Komet Dental, Germany). Burs were replaced after every 20 preparations to maintain consistent cutting efficiency. A second operator verified preparation dimensions using a digital caliper to confirm standardization.

Two bonding substrates were investigated: tooth substrate and resin-composite. The resin composite condition in the cavity simulated an abutment containing an existing resin composite restoration or elevated margins or cavity floor. For the tooth substrate group, no additional restorative material was placed after cavity preparation. For the resin- composite substrate group, cavities were initially roughened with burs (Intensive Cerinlay burs (#011)) under water-spray cooling. Cavities were then restored with a direct resin composite using a standardized adhesive protocol: etching with 37% phosphoric acid (enamel: 30 s; dentin: 15 s), followed by rinsing with a water spray for 30 s and gentle air drying; application of a primer (OptiBond FL Primer, Kerr, USA) with a microbrush for 15 s; application of an adhesive resin (OptiBond FL Adhesive, Kerr, USA) for 15 s, followed by gentle air spray for 15 s; photo-polymerization for 40 s with a calibrated photo-polymerization unit; placement of resin composite (Ceram.x Spectra STHV; Dentsply Sirona) in increments not exceeding 2 mm. Each increment was photo-polymerized for 30 s.

### Framework fabrication

All experimental models were digitized with a laboratory scanner (Medit T710, Seoul, South Korea). The scan data were exported as STL files and used to design the IRFDPs.

Each IRFDP was designed (exocad DentalCAD, exocad GmbH, Darmstadt, Germany) to replace a missing premolar. The pontic was constructed without contact with the model base to avoid artificial support during mechanical testing. Connector dimensions were standardized to a minimum cross-sectional area of 9 mm² (3 mm width × 3 mm height) to ensure uniform structural conditions across all specimens, within the clinically recommended range for posterior zirconia FDPs, and to allow sufficient mechanical stability while maintaining sensitivity to material-dependent differences in fracture behavior [3, 7].

After digital design, 160 IRFDPs were fabricated (PrograMill PM7, Ivoclar AG) and randomly assigned to four zirconia materials (*n* = 40 pr material) (Table 1):

1. IPS e.max ZirCAD LT (Ivoclar).
2. Katana YML (Yttria Multi Layered; Kuraray Noritake).
3. Katana HTML Plus (High Translucent Multi Layered Plus; Kuraray Noritake).
4. Katana STML (Super Translucent Multi Layered; Kuraray Noritake).

All restorations were milled according to the manufacturer’s recommendations.

**Table 1:** Overview of the materials used in the study

Material	Manufacturer	Chemical Composition
IPS e.max ZirCAD LT	Ivoclar AG	Zirconium oxide (ZrO_2_) 88.0–95.5 wt%, yttrium oxide (Y_2_O_3_) > 4.5 - ≤ 6.0 wt%, hafnium oxide (HfO_2_) ≤ 5.0 wt%, aluminium oxide (Al_2_O_3_) ≤ 1.0 wt%, other oxides for colouring ≤ 1.0 wt%
Katana YML (Yttria Multi Layered)	Kuraray Noritake Dental, Japan	Zirconium dioxide (ZrO_2_), yttrium oxide (Y_2_O_3_) (not specified)
Katana HTML Plus (High Translucent Multi Layered Plus)	Kuraray Noritake Dental, Japan	Zirconium dioxide (ZrO_2_) 80–95%, yttrium oxide (Y_2_O_3_) 3–15%
Katana STML (Super Translucent Multi Layered)	Kuraray Noritake Dental, Japan	Zirconium dioxide (ZrO_2_) 80–95%, yttrium oxide (Y_2_O_3_) 3–15%
OptiBond FL Primer	Kerr Corp, Orange, USA	2-hydroxyethyl methacrylate (HEMA) 10–30 wt%, ethanol 30–60 wt%, water 10–30 wt%, glycerol-phosphate dimethacrylate (GPDM) < 10 wt%, photoinitiators and stabilizers < 1 wt%
OptiBond FL Adhesive	Kerr Corp, Orange, USA	Barium-alumino-borosilicate glass filler 30–60 wt%, glycerol-phosphate dimethacrylate (GPDM) 10–30 wt%, 2-hydroxyethyl methacrylate (HEMA) 5–20 wt%, bisphenol-A glycidyl dimethacrylate (Bis-GMA) 5–20 wt%, photoinitiators and amine accelerators < 1 wt%, stabilizers < 1 wt%
Ceram.x Spectra ST HV	Dentsply Sirona	Barium-alumino-fluoro-borosilicate glass and silica fillers ~ 73–77 wt%, urethane dimethacrylate (UDMA) 10–20 wt%, ethoxylated bisphenol-A dimethacrylate (Bis-EMA) 5–15 wt%, triethylene glycol dimethacrylate (TEGDMA) < 10 wt%, camphorquinone < 1 wt%, amine photoinitiators < 1 wt%, pigments and stabilizers < 1 wt%
Panavia 21	Kuraray Noritake Dental, Japan	Catalyst paste: 10-methacryloyloxydecyl dihydrogen phosphate (10-MDP), hydrophobic aromatic dimethacrylate, hydrophobic aliphatic dimethacrylate, silanated silica filler, colloidal silica, catalysts. Universal paste: hydrophobic aromatic and aliphatic dimethacrylates, silanated titanium oxide and barium glass fillers, catalysts, accelerators, pigments
Clearfil Ceramic Primer Plus	Kuraray Noritake Dental, Japan	Silane coupling agent, 10-MDP (10-methacryloyloxydecyl dihydrogen phosphate), ethanol
3 M Rocatec Soft	3 M, St. Paul, MN, USA	Aluminium oxide (Al_2_O_3_): 98–100 wt% Sodium oxide (Na_2_O): < 1 wt%

### Cementation

For the tooth substrate group, enamel was etched with 37% phosphoric acid for 30 s, and tooth for 15 s. The surfaces were rinsed thoroughly with a water spray for 30 s and then gently air-dried. Subsequently, primer (ED Primer A and B, (Panavia 21 system, Kuraray Noritake) was mixed in a 1:1 ratio and applied to the prepared surfaces with a microbrush for 60 s, followed by gentle air-drying.

For the resin composite substrate group, the cavity surfaces were air-abraded with 30 μm aluminum oxide particles at 2.5 bar from a distance of 10 mm for 5 s. The surfaces were rinsed with a water spray for 30 s and air-dried. Clearfil Ceramic Primer Plus (Kuraray Noritake) was then applied for 60 s and air-dried for an additional 60 s before cementation.

Before cementation, all zirconia restorations were cleaned (Ivoclean, Ivoclar) for 30 s per manufacturer’s instructions. The paste was removed with a water spray, and the restorations were air-dried. The adhesive surfaces were then air-abraded with 30 μm aluminum oxide particles (3 M Rocatec Soft, 3 M, St. Paul, MN, USA) at 2.5 bar from a standardized distance of 10 mm for 20 s. After air-abrasion, the restorations were cleaned in distilled water ultrasonically for 5 min and air-dried. A ceramic primer (Clearfil Ceramic Primer Plus, Kuraray Noritake) was applied to the intaglio surface for 60 s and gently air-dried for an additional 60 s. Cementation was performed with a dual-polymerizd resin cement (Panavia 21, Kuraray Noritake) (Table 1). The base and catalyst pastes were mixed in a 1:1 ratio for 30 s according to the manufacturer’s instructions and applied to the restoration. The IRFDPs were seated under a constant load of 2.5 N using a custom loading device with calibrated weights to standardize the cementation procedure. After seating, excess cement was removed. An oxygen-inhibiting gel (Oxyguard, Kuraray Noritake) was applied to the margins for 7 min to ensure complete polymerization.

### Thermo-mechanical aging

Specimens assigned to the aging group were subjected to thermomechanical loading in a chewing simulator (Zurich Chewing Simulator, Zurich, Switzerland) [17–19]. The aging protocol consisted of 1,200,000 loading cycles at 50 N, applied at 1.67 Hz over 9 days. Simultaneously, thermocycling was performed between 5 °C and 55 °C in distilled water to simulate intraoral temperature fluctuations. The load was applied perpendicular to the occlusal surface of the centrally positioned pontic, at the main fossa, using a stainless-steel antagonist sphere with a diameter of 3 mm*.*

### Monotonic test and failure analysis

After thermo-mechanical aging, all aged specimens were visually inspected for debonding, cracks, and other premature failures. Specimens were then subjected to monotonic load-to-fracture testing to determine the maximum fracture load.

Mechanical testing was performed on a universal testing machine (Zwick/Roell Z050; Zwick, Ulm, Germany). A compressive load was applied perpendicular to the occlusal surface of the pontic using a stainless-steel loading piston. The load was applied at a crosshead speed of 1 mm/min until failure. The maximum load at fracture (N) was recorded for each specimen.

After fracture testing, failure modes were analyzed using a digital microscope (VHX-7000; Keyence, Osaka, Japan) at 20× magnification. Digital images were obtained for documentation. Failure patterns were categorized according to their clinical reparability. Reparable failures were defined as failures not involving structural fracture of the connector or extensive material loss. These included partial debonding or the presence of a visible crack line in the zirconia framework, where the restoration remained structurally intact and could potentially be corrected through surface treatment, reconditioning, or rebonding procedures. Irreparable failures were defined as failures involving complete debonding, connector fracture, cohesive framework fracture with material loss (chipping), or mixed failures combining debonding and connector fracture. In such cases, structural integrity was compromised, rendering predictable repair unfeasible and necessitating replacement of the restoration.

### Statistical analysis

The power of the study was calculated using G*Power software (Version 3.1.9.6, Fraz Faul, Universität Kiel, Kiel, Germany), considering the sample size (*n* = 10 per group), the mean values obtained for each experimental condition, and a common standard deviation (SD) for each condition. A significance level (α) of 0.05 was applied. For the dry condition, a common SD of 390 N was used, resulting in a statistical power of 0.9681 and an effect size of 0.7038. For the aging condition, a common SD of 343.was considered, yielding a power of 0.9706 and an effect size of 0.8695. These results indicate that the study had a high probability of detecting statistically significant differences among the groups under both conditions.

Statistical analyses were performed using SPSS (version 21; IBM Analytics, NY, USA) and Statistix 10 (Analytical Software, FL, USA).

Fracture load data were assessed for normality (Shapiro-Wilk test) and homogeneity of variance (Levene’s test). As assumptions were satisfied (*p* > 0.05), parametric analyses were applied. Two-way ANOVA was used to evaluate the effects of material and substrate, and their interaction, separately for dry and aged conditions. Post hoc comparisons were performed using Tukey’s test (α = 0.05). Additionally, independent-samples Student’s t-tests were performed to compare the dry and aged conditions within each material–substrate combination (α = 0.05).

Weibull analysis was conducted for each material–substrate combination (SuperSMITH Weibull 4.0k-32; Wes Fulton, USA) to determine the Weibull modulus (m) and characteristic strength (σ₀). Given the subgroup size, Weibull parameters were interpreted descriptively using 95% confidence intervals.

Failure modes were analyzed descriptively and expressed as absolute numbers and percentages.

## Results

Two-way ANOVA revealed significant effects of material (F = 4.70; *p* = 0.0047) and substrate type (F = 5.34; *p* = 0.0237), with a significant interaction between material and substrate type (F = 6.75; *p* = 0.0004) (Table 2).

**Table 2:** Results of two-way ANOVA tests

Source	DF	SS	MS	F	*P*
Dry					
Material	3	2,142,110	714,037	4.70	0.0047
Substrate	1	811,642	811,642	5.34	0.0237
Material * Substrate	3	3,075,529	1,025,176	6.75	0.0004
Error	72	1.094E + 07	151,990		
Total	79	1.697E + 07			
Aging					
Material	3	3,801,396	1,267,132	10.94	0.0000
Substrate	1	145,630	145,630	1.26	0.2660
Material * Substrate	3	3,170,284	1,056,761	9.12	0.0000
Error	72	8,342,205	115,864		
Total	79	1.546E + 07			

Within the resin composite substrate, YML (1406 ± 386) showed a significantly higher fracture load (N) than STML (553 ± 246). HTML (1142 ± 249) also presented a significantly higher fracture load than STML. Thus, STML exhibited significantly lower fracture resistance than both YML and HTML when bonded to resin composite. Within the tooth substrate, no statistically significant differences were observed among the materials, despite numerical differences in mean fracture load. ZirCAD LT (1550 ± 369) showed the highest mean value but it did not differ statistically from other materials when bonded to tooth substrates. Across both substrates, STML showed a significant reduction in fracture load when bonded to resin composite compared with tooth substrates, while YML exhibited a higher fracture load when IRFDPs were bonded to resin composite than on tooth substare. ZirCAD LT and HTML showed no significant substrate-dependent differences. ZirCAD LT and HTML behaved as intermediate groups overall, as they did not differ significantly from the higher- or lower-performing materials across substrates. YML demonstrated superior performance over STML in the composite condition, while STML showed the lowest fracture resistance under composite bonding. The remaining material-substrate combinations were statistically comparable (Table 3).

**Table 3:** Mean (N), standard deviation (SD), and Weibull modulus (*m*) for load-bearing capacity

Material	Substrate	Load-bearing capacity (N) (Mean ± SD)*		Weibull modulus (95% CI)**	
		Dry	Aging	Dry	Aging
HTML	Tooth	1190 ± 450 ^Aa^	1727 ± 351 ^Ab^	3.14 ^AB^ (1.89–5.20)	5.65 ^A^ (3.44–9.30)
	Composite	1142 ± 249 ^Aa^	1274 ± 375 ^ABCa^	5.29^A^ (3.27–8.54)	4.24 ^A^ (2.57–6.99)
STML	Tooth	1199 ± 318 ^Aa^	1109 ± 274 ^BCDa^	4.45 ^AB^ (2.75–7.22)	4.54 ^A^ (2.85–7.24)
	Composite	553 ± 246 ^Ba^	718 ± 292 ^Da^	2.52 ^AB^ (1.59–3.99)	2.93 ^A^ (1.73–4.98)
YML	Tooth	1043 ± 576 ^ABa^	831 ± 312 ^CDa^	1.84 ^B^ (1.07–3.16)	3.14 ^A^ (1.84–5.34)
	Composite	1406 ± 386 ^Aa^	1381 ± 376 ^ABa^	4.77 ^AB^ (2.84–8.01)	4.24 ^A^ (2.65–6.82)
ZirCAD LT	Tooth	1550 ± 369 ^Aa^	1076 ± 449 ^BCDb^	5.23 ^AB^ (3.16–8.67)	2.83 ^A^ (1.69–4.73)
	Composite	1075 ± 416 ^ABa^	1028 ± 277 ^BCDa^	2.93 ^AB^ (1.83–4.70)	4.29 ^A^ (2.66–6.93)

* Different uppercase letters indicate statistical differences among material–substrate combinations within the same column as determined by Two-way ANOVA and Tukey’s post-hoc tests (α= 0.05). Different lowercase letters indicate statistically significant differences between Dry and Aging within the same material–substrate combination, as determined by independent-samples Student’s t-tests (α = 0.05). Different lowercase letters indicate statistically significant differences between Dry and Aging within the same material–substrate combination, as determined by independent-samples Student’s t-tests (α= 0.05).** Different letters indicate statistical differences among material–substrate combinations within the same

** Different letters indicate statistical differences among material–substrate combinations within the same column according to Weibull analysis using the maximum likelihood estimation method, with overlapping 95% confidence intervals indicating statistical similarities

**Fig. 2:**
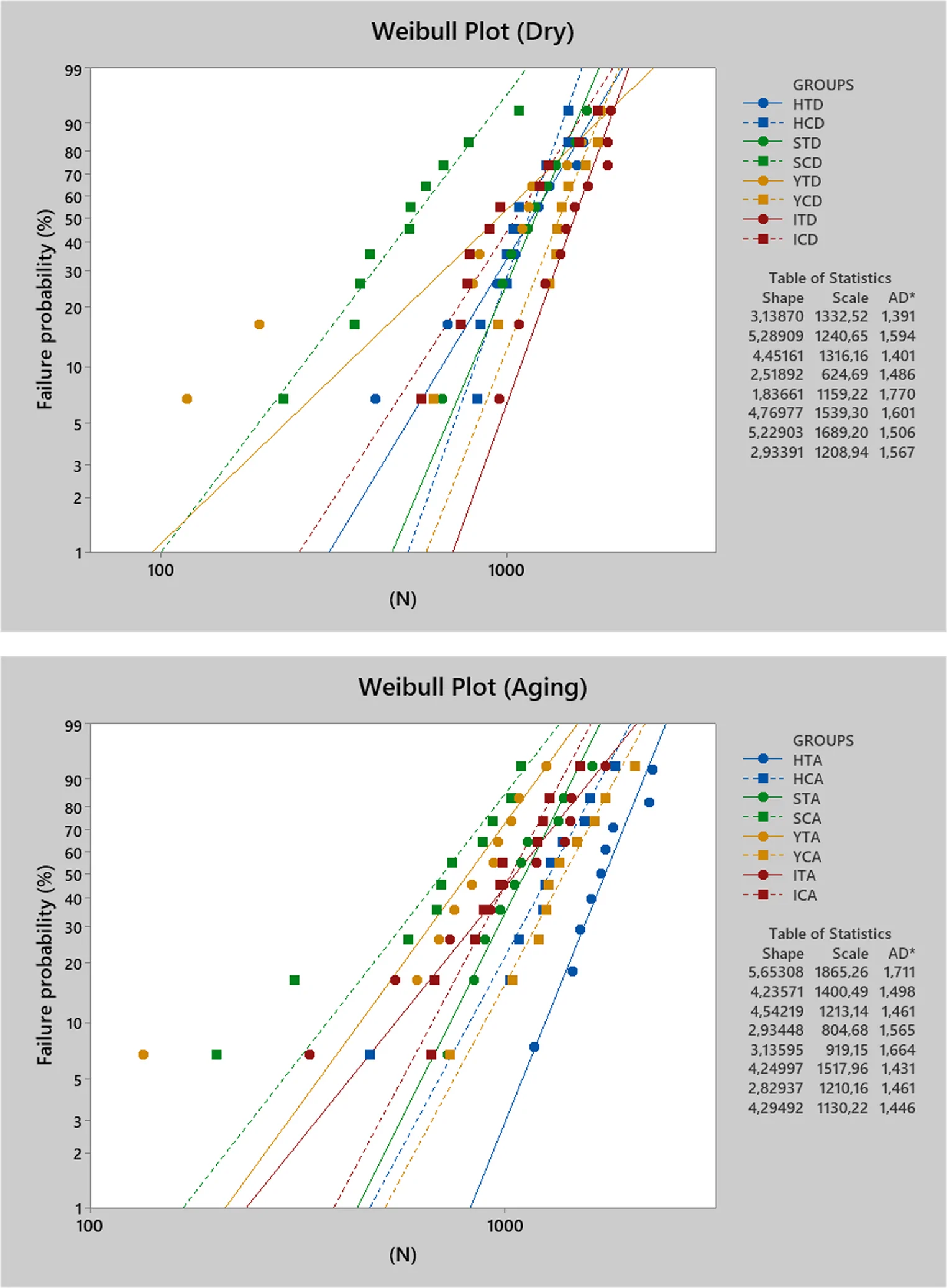
Weibull probability plots showing the failure probability of load-bearing capacity for all surface substrate-materials combinations. The plot compares the different groups on the estimated characteristic strength (η) and Weibull modulus (β)

 Under dry conditions, HTML on composite exhibited a significantly higher Weibull modulus than YML on tooth, indicating greater structural reliability and lower variability in fracture load for this group (Fig. 2). All other material-substrate combinations showed intermediate Weibull modulus values and did not differ statistically from either HTML on composite or YML on tooth.

**Table 4:** Distribution of failure modes (%) for each zirconia material and substrate under dry and thermo-mechanical aging conditions. Failures were categorized as reparable (partial debonding or crack formation without connector fracture) or irreparable (complete debonding, connector fracture, cohesive bulk fracture, or mixed failure). Percentages represent the proportion of specimens within each material-substrate group

Material	Substrate	Failure Type (%)			
		Dry		Aging	
		Reparable	Irreparable	Reparable	Irreparable
HTML	Tooth	50	50	22.2	77.8
	Composite	30	70	60	40
STML	Tooth	60	40	40	60
	Composite	70	30	80	20
YML	Tooth	60	40	60	40
	Composite	50	50	30	70
ZirCAD LT	Tooth	60	40	90	10
	Composite	60	40	80	20

Two-way ANOVA revealed statistically significant effects of material (F = 4.70; *p* = 0.0047) and substrate (F = 5.34; *p* = 0.0237), as well as a significant interaction between material and substrate (F = 6.75; *p* = 0.0004), indicating that the effect of substrate on fracture load depended on the zirconia material. HTML bonded to tooth (1727 ± 351) demonstrated a significantly higher fracture load than STML bonded to resin composite (718 ± 292), YML bonded to tooth substrate (831 ± 312), and ZirCAD LT bonded to tooth (1076 ± 449 N) and composite (1028 ± 277). YML bonded to composite (1381 ± 376) also showed a significantly higher fracture load than STML bonded to composite and YML bonded to tooth.

STML bonded to tooth (1109 ± 274) exhibited a significantly higher fracture load than STML bonded to composite. HTML bonded to composite (1274 ± 375), YML bonded to composite, and STML bonded to tooth showed intermediate values and did not differ significantly from several higher- or lower-performing groups. ZirCAD LT on both substrates and YML bonded to tooth showed no significant differences among themselves and were statistically comparable to multiple other groups.

For the aging condition, no statistically significant differences in Weibull modulus were observed across the material-substrate combinations. All groups showed comparable reliability, with overlapping confidence intervals.

Visual inspection after thermo-mechanical aging revealed no debonding, cracks, or other premature failures before monotonic testing.

**Fig. 3:**
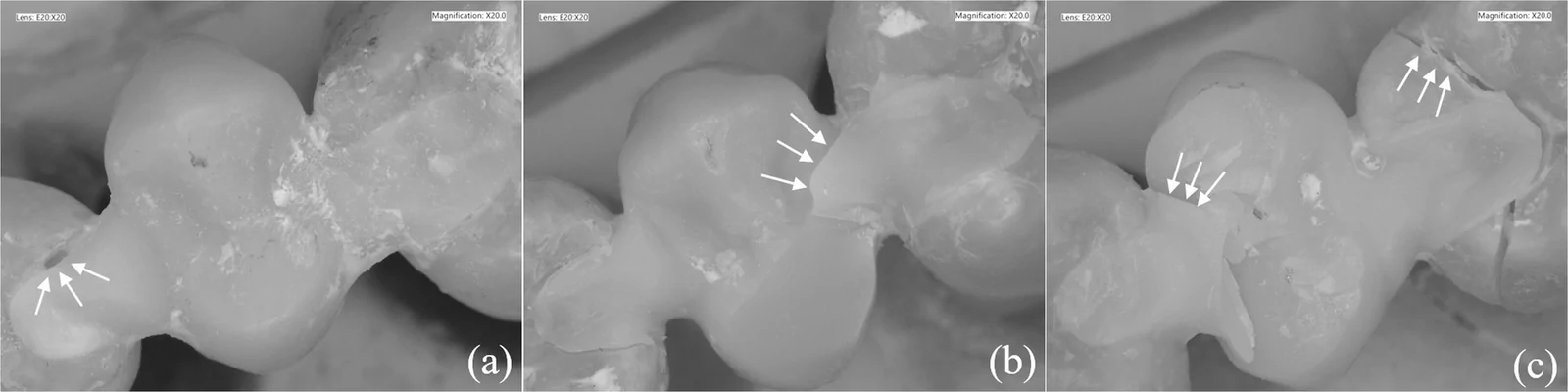
Representative images for the types of failures (**a**) partial debonding; (**b**) crack formation without connector fracture; and (**c**) connector fracture and cohesive bulk fracture

Under dry conditions, the distribution of reparable and irreparable failures varied by material and substrate (Table 4, Fig. 3). Most material-substrate combinations showed a predominance of reparable failures (STML-tooth, STML-composite, YML-tooth, ZirCAD LT-tooth, ZirCAD LT-tooth), while certain groups had a higher proportion of irreparable fractures (HTML-composite). No consistent overall trend favoring a specific substrate was observed across all materials in the dry condition. After thermo-mechanical aging, the failure distribution changed in several groups. Some material-substrate combinations exhibited an increased proportion of reparable failures (HTML-composite, STML–composite, ZirCAD LT-tooth, ZirCAD LT-composite), whereas others shifted toward more irreparable failures (HTML-tooth, YML-composite). Overall, failure behavior after aging remained material-dependent, with no uniform substrate effect across all zirconia types.

## Discussion

 The tested materials in this study represent distinct zirconia formulations. IPS e.max ZirCAD LT and Katana HTML Plus are predominantly 3Y-TZP, Katana STML contains a higher yttria content to enhance translucency, and Katana YML combines layers containing approximately 3 to 5 mol% yttria to create a translucency gradient also reinforcing the strength [8, 9].

The aim of the present study was to evaluate the influence of zirconia type, bonded substrate (tooth substrate or resin composite), and thermo-mechanical aging on the load-bearing capacity and Weibull characteristics of IRFDPs. The first null hypothesis, stating that zirconia type would not influence the load-bearing capacity and Weibull characteristics of IRFDPs, was rejected since significant differences among materials were observed. The second null hypothesis, assuming that the bonded substrate would not influence the load-bearing capacity and Weibull characteristics, was partially rejected because the substrate type affected fracture load under dry conditions but not after aging. The third null hypothesis, that aging would not influence load-bearing capacity and Weibull characteristics, was also partially rejected as aging affected fracture load values but did not significantly affect Weibull reliability.

The differences observed among the tested zirconia materials can be explained primarily based on their microstructural composition and yttria content. Conventional zirconia, such as IPS e.max ZirCAD LT, is a 3Y-TZP material with a predominantly tetragonal phase, which provides high flexural strength and fracture toughness through transformation toughening mechanisms [6, 7]. Increasing the yttria content stabilizes the cubic phase, improving translucency but simultaneously reducing crack-arrest capability and transformation toughening, which results in lower mechanical properties [6, 7]. This inverse relationship between translucency and strength has been consistently reported in previous laboratory studies [8, 9]. Katana HTML, for example, is principally based on 3Y-TZP zirconia and presents a multilayer structure that is mainly achieved through pigmentation rather than major compositional differences across layers [9]. In contrast, Katana STML contains higher yttria concentrations, resulting in an increased cubic phase fraction and therefore enhanced translucency but reduced mechanical performance [8]. Katana YML represents a compositionally graded zirconia combining approximately 3 mol% yttria in the cervical region and up to 5 mol% yttria in the incisal region, creating a strength, translucency gradient within the same blank [6].

Despite differences in mean fracture load among materials, Weibull analysis offered insight into the reliability of tested zirconia systems. Under dry conditions, higher Weibull modulus values for some combinations (e.g., HTML bonded to composite) indicated predictable failure, while lower values (e.g., YML bonded to tooth) showed greater variability [11]. Most groups had overlapping confidence intervals, suggesting limited, inconsistent reliability differences. After aging, Weibull modulus values became more uniform across groups, indicating aging reduces reliability differences and leads to a more consistent failure behavior, possibly due to fatigue mechanisms [17–19]. Clinically, materials with higher Weibull modulus may perform more predictably, even with similar fracture loads [11]. However, small subgroup size and overlapping confidence intervals mean Weibull results are suggestive rather than definitive.

The bonded substrate also influenced the mechanical behavior of the restorations. Under dry conditions, IRFDPs bonded to tooth showed slightly higher fracture loads compared with those bonded to composite. One possible explanation may be the presence of enamel margins surrounding the tooth cavity preparations, which could contribute to a more predictable adhesive interface. Adhesion to enamel is generally considered more reliable than adhesion to tooth due to its highly mineralized and homogeneous structure [12]. In addition, preservation of the dentin-enamel junction may improve biomechanical behavior, as the elastic modulus mismatch between enamel and dentin can help arrest crack propagation and distribute stresses more effectively [16, 20]. A gradient of elastic modulus has been reported from the enamel surface toward the dentin-enamel junction, which may further enhance crack resistance in natural teeth [21, 22]. Although the present study compared tooth and composite substrates rather than enamel and dentin directly, these biomechanical principles support the interpretation that the surrounding tooth structure may influence stress distribution within the restoration. Composite substrates also demonstrated predictable mechanical behavior. Resin composite may provide a relatively homogeneous and elastic interface that contributes to stress distribution within the tooth-restoration complex [23]. Furthermore, increased loss of tooth structure has been associated with reduced fracture resistance of restored teeth [24, 25], emphasizing the importance of minimally invasive preparation designs. After thermo-mechanical aging, however, no significant differences were observed between tooth and composite substrates.

Thermo-mechanical aging produced only limited reductions in fracture load. The aging protocol simulated the cyclic loading and thermal stresses experienced during clinical service. Previous studies have shown that fracture loads of adhesively bonded ceramic restorations frequently exceed physiological chewing forces and may even surpass parafunctional loads [26]. Zirconia ceramics are known for their high resistance to fatigue and crack propagation compared with other dental ceramics [4]. In addition, fatigue studies of adhesively bonded restorations have reported high survival rates when adequate bonding and preservation of tooth structure are present [17–19]. The relatively small reduction in fracture load observed in the present study, therefore, suggests good mechanical stability of zirconia IRFDPs under simulated functional conditions. Clinically, these findings indicate that zirconia materials may provide sufficient load-bearing capacity in posterior regions when appropriate design parameters and adhesive protocols are applied [2, 3].

 Failure mode analysis provided additional information beyond fracture load values. Reparable failures included partial debonding at the retainer–adhesive interface or crack formation without connector fracture, whereas irreparable failures comprised complete debonding, connector or cohesive framework fracture, and mixed failures. After aging, irreparable failures predominated in the HTML-tooth and YML-composite groups, while aged STML-composite and ZirCAD LT groups showed mainly reparable failures. This material-dependent pattern may indicate that the critical failure site shifts between the adhesive/retainer region and the zirconia framework, depending on the material-substrate combination. Connector fractures are clinically important because connector geometry and zirconia microstructure influence stress concentration and fracture behavior [3, 7], whereas adhesive failures are more closely related to substrate and bonding conditions [2, 12]. Pontic failures were not recorded as a separate category. Hence, conclusions regarding pontic-specific complications cannot be drawn from the present data.

 The use of third molars in this study allowed for elimination of possible caries-affected enamel or dentin which could be the case clinically. Clinical abutments may also include enamel, dentin, and/or existing restorative materials at the same time, while in this study experimental groups represented tooth and composite substrates separately. Consequently, the findings may not directly reflect heavily restored abutments, in which reduced residual tooth structure and multiple adhesive interfaces may affect load distribution and bonding durability. Only one connector geometry was evaluated representing the ideal connector diameter. Therefore, the findings may not apply to other prosthesis designs which is also sometimes dictated by the height of the abutments and the periodontal support [27–29]. Nevertheless, ranking of the materials in this study should be considered prior to clinical trials especially with multilayer zirconia IRFDPs. The findings obtained from the performance of IRFDPs when bonded only onto resin composite substrate relates to increasingly applied proximal box elevation, expanding the indication of IRFDPs bonded to this substrate. Accordingly, clinicals trials on survival of IRFDPs should report on the type of substrate along with the zirconia type and conditioning protocols.

## Conclusions

From this study, the following can be concluded:

1. The load-bearing capacity of inlay-retained fixed dental prostheses was significantly influenced by the type of zirconia material, indicating that differences in yttria content and microstructural composition affect the mechanical performance of these restorations.
2. 3Y-TZP zirconia materials demonstrated higher fracture load values compared to the more translucent zirconia materials with higher yttria content.
3. The bonded substrate affected the fracture load under dry conditions, with restorations bonded to tooth substrate showing slightly higher load-bearing capacity, but after thermo-mechanical aging no significant differences between tooth and composite substrates were observed.
4. Thermo-mechanical aging resulted in only limited reductions in fracture load, suggesting that zirconia IRFDPs exhibit stable mechanical performance under simulated functional conditions, providing that irreparable fractures were more common in high-translucent multilayer zirconia bonded to composite substrates.
5. Weibull analysis revealed comparable reliability among the tested zirconia materials, indicating similar defect distribution and structural predictability despite differences in mean fracture load.

## Data Availability

Datasets used and/or analyzed during this study are available from the authors upon reasonable request.
